# The poetry of senses: exploring semantic mediation in timbre-aroma correspondences

**DOI:** 10.3389/fpsyg.2025.1520046

**Published:** 2025-02-25

**Authors:** Asterios Zacharakis

**Affiliations:** School of Music Studies, Aristotle University of Thessaloniki, Thessaloniki, Greece

**Keywords:** semantic mediation, timbre, aroma, cross-modal correspondences, audition, olfaction

## Abstract

Cross-modal correspondences between audition and olfaction have received relatively less attention compared to other modality pairs. This study expands on previous work regarding timbre-aroma correspondences by examining the semantic mediation hypothesis, according to which cross-modal correspondences may be partly explained by the existence of common semantic qualities. In a behavioral experiment, 26 musically trained participants rated 26 complex synthetic tones and 12 aromatic stimuli across two separate blocks using a common set of semantic scales. The analysis of semantic variables identified a largely consistent organization for both modalities, condensing into three prominent clusters: [*bright, fresh, ethereal*], [*sharp, metallic*], and [*full, rich, warm*]. Furthermore, distances between stimuli derived from semantic ratings and optimized through a genetic algorithm exhibited a strong correlation with previously estimated ground-truth distances of direct cross-modal associations. Additionally, the stimulus configuration within the semantic space generated through Multidimensional Scaling analysis exhibited notable commonalities with the organization of stimuli derived from direct timbre-aroma correspondences. Overall, this study provides compelling evidence that semantic mediation plays a significant role in shaping cross-modal correspondences between auditory and olfactory stimuli, paving the way for further exploration of the underlying semantic dimensions that connect these two modalities.

## 1 Introduction

The growing research interest in multisensory perception over the past few decades has offered compelling insights into the complex interconnectedness of sensory systems. On the one hand, our understanding of how sensory stimuli processed through distinct sensory pathways may correspond has deepened. On the other, studies have shown that cross-modal congruencies—or lack thereof—have a noticeable influence on how we interpret and engage with the world through our senses.

It has been suggested that the mechanisms accounting for the observed cross-modal associations fall into the following categories: associative learning; activation of common brain regions through supramodal properties (e.g., intensity); common affective responses; and shared semantic properties (Spence, 2011, 2020a). The current study will focus on the latter, building on recent findings on auditory-olfactory associations by Zacharakis (2024). Besides this cross-modal pair being arguably the least explored to date, the apparent ineffability characterizing these two senses constitutes an additional challenge in assessing possible semantic mediation in their crosstalk. It has been suggested that the chemical senses (i.e., gustation and olfaction) are the most difficult to express verbally, with audition falling in between these two and the more easily articulated senses of touch and vision (Winter, 2019). To complicate matters further, most descriptors for olfaction overwhelmingly derive from categories related to the source of the stimulus (e.g., floral, fruity, yeasty, etc.) (e.g., Cain et al., 1998; Burlingame et al., 2004; Zarzo and Stanton, 2009; Kaeppler and Mueller, 2013; Hörberg et al., 2022). This is also somewhat true for audition where descriptions such as “percussive,” “tinny,” “electronic,” or “brassy” are commonly used (e.g., Zacharakis et al., 2014).

The dominance of source-related descriptors in olfaction reduces the likelihood of semantic overlap with audition, especially since explicit olfactory metaphors are absent from the cognitive linguistics of timbre literature. Nevertheless, abstract descriptions of sound impressions are quite common and very often take the form of synaesthetic metaphors such as “bright,” “gloomy,” “rough,” or “smooth,” (e.g., Zacharakis et al., 2014; Wallmark and Kendall, 2018; Saitis and Weinzierl, 2019). Similarly, abstract descriptions of olfactory qualities have also been noted in various studies (e.g., Velasco et al., 2014; Deroy et al., 2013; Hörberg et al., 2022) suggesting the potential for shared semantic properties between these two sensory modalities^1^. Indeed, cross-modal loans such as *bright, warm, sharp* or *sweet* along with source-material properties like *metallic* or *woody*, are well established timbral descriptors (for an overview, see Wallmark and Kendall, 2018; Saitis and Weinzierl, 2019) and while less frequent, also appear in scent descriptions (von Hornbostel, 1931; Cohen, 1934; Hörberg et al., 2022; Spence, 2021; Spence et al., 2021a).

Although olfactory-auditory crosstalk has received limited attention to date, research into both cross-modal associations and interactions between these two senses is gaining momentum. The former aims to enhance our understanding of potential correspondences between different sensory modalities, while the latter applies this understanding to investigate multisensory interactions shaped by congruent or incongruent relationships. Earlier studies on olfactory-auditory relationships have revealed associations between certain odors and aspects of pitch or instrument family classes (Belkin et al., 1997; Crisinel and Spence, 2012). More recently, Mesz et al. (2023) identified links between higher-level musical characteristics such as articulation, ambitus or dissonance through a musical improvisation task inspired by olfactory stimulation. Within the context of multisensory interplay, evidence suggests that background noise can influence the appreciation of olfactory experiences, while the effects of background music remain less clear (e.g., Velasco et al., 2014, for a review see Spence, 2014). Overall, advances in our understanding of the cross-modality between sounds and scents have led researchers to advocate for the potential applications of such relationships, particularly in the crafting of multisensory experiences (Mattila and Wirtz, 2001; Seo et al., 2013; Spence, 2021), as well as in sonic branding (e.g., Mahdavi et al., 2020; Spence and Keller, 2024; Spence et al., 2024; Rodŕıguez et al., 2024; Velasco and Spence, 2024).

Bringing a closer focus on timbre, recent work by Zacharakis (2024) has identified above-chance associations between aromatic oils and synthetic timbres, thereby expanding the established associations between aromas and pitch or basic musical instrument categories (Crisinel and Spence, 2012). The study developed a formula to convert the distributions of correspondences between sounds and scents into direct distances. These data provide a valuable opportunity to explore the potential connection between shared cross-modal qualia –as expressed through lexical means– and the identified cross-modal relationships. The primary question here would be whether the semantics of timbre and scent communicate common underlying qualities that could partly account for the observed cross-modal associations or differences. To this end, a common set of abstract descriptors was selected to evaluate the aromatic and sound stimuli from Zacharakis (2024) in two separate experiments utilizing a within-subjects design.

The following section will present the rationale behind the descriptor selection (Section 2.3) and provide details about stimulus creation and the experimental procedure. The results section will first compare the structure of the semantic variables across olfaction and audition. It will then offer a global perspective by comparing the direct distances between cross-modal stimuli (ground truth) with indirect distance estimations stemming from semantic scale vectors with optimized weights. Finally, it will focus on examining the distances between specific sound-scent pairs, as represented in a common timbral-aromatic space derived from Multidimensional Scaling (MDS) analysis of semantic differences. The paper will conclude by discussing the implications of these findings, highlighting their contribution to a deeper understanding of cross-modal associations between timbre and aroma.

## 2 Materials and methods

### 2.1 Auditory and olfactory stimuli

The stimuli used in this study were identical to the ones used in Zacharakis (2024). The sound stimulus set consisted of 26 complex synthetic tones that were created through various combinations of sound synthesis (frequency modulation, amplitude modulation, wavetable, additive, and granular synthesis) and/or sound processing (filtering, reverb, delay, phasing, etc.) implemented using Ableton Live. This approach was preferred over the use of familiar instrumental timbres to mitigate potential confounding factors in auditory-olfactory associations caused by source-cause categorization (Siedenburg, 2017). In addition, it allowed greater flexibility in designing timbres that align with the acoustical correlates of scent suggested by the literature (Crisinel and Spence, 2012; Crisinel et al., 2013; Deroy et al., 2013; Spence, 2021). Stimulus duration ranged from 6 to 12 s, while the pitch also varied, ranging from G2 (98 Hz) to G5 (784 Hz). Several of the stimuli comprised tone combinations and complex temporal fluctuations. For further details on the sound stimulus design, readers are directed to the *Sound stimuli and apparatus* subsection in Zacharakis (2024).

The sound stimuli were delivered via a MacBook Pro laptop (Apple Computer, Inc., Cupertino, CA), using a custom-built graphical user interface in Max/MSP for stimulus playback and data acquisition. Listeners were presented with the sound stimuli binaurally using Beyerdynamic DT-880 PRO headphones (250 Ohm). Loudness was equalized across all stimuli at a comfortable playback level through informal listening tests. This resulted in RMS levels between 65 and 75 dB SPL (A-weighted, slow response). The sound stimuli are available in the Supplementary material. At this point, it should be noted that the sound labels employed throughout the manuscript derive directly from the names of their intended aromatic counterparts (for more details see Zacharakis, 2024). Given that these sounds lack a physical source, this methodology was considered more advantageous than simply labeling the sound stimuli as S1–S26. This approach allows the reader to be informed about the intended aromatic target corresponding to each sound.

The twelve aromatic stimuli were introduced using small glass bottles (5 ml) sealed with a plastic screw cap, each containing a piece of cotton on the inside. The cotton in each bottle was moistened with three drops from a selection of 11 different aromatic oils, namely, vanilla, honey, caramel, cinnamon, coffee, (black) pepper, lemon, lemon blossom, pomegranate, melon, and cherry. The 12th aromatic stimulus, tobacco, was presented through leaves enclosed in a small plastic container with a screw cap (8 ml). In contrast to Zacharakis (2024), the presented aromas were labeled only with code numbers from 1 to 12 (Ward et al., 2022) thus not explicitly disclosing the identity of each aroma (with a possible exception of tobacco, whose leaves' appearance must have been familiar for most participants). The data acquisition was made through a Max/MSP graphical user interface equivalent to the one used for the sound stimuli presentation.

### 2.2 Participants

A convenience sample of 26 participants (mean age: 23.6 years, age range: 19–56 years, 14 females) took part in the experiment. The majority were students at the Aristotle University of Thessaloniki who gave their informed consent and received course credit compensation for their participation. The responses from two participants in the aroma evaluation experiment were lost, leaving a total of 24 raters for this experimental component.

### 2.3 Semantic descriptors

The selection of semantic scales was based on the inclusion of terms that could serve as common descriptors for both olfactory and auditory stimuli. The verbal description of sounds and scents often facilitates communication on either source identification or classification (e.g., smells like wet soil, sounds like rumbling thunder, etc.). Such verbalisations are unlikely to feature much overlap. However, as also noted in the introduction, there appears to be quite some overlap in descriptions that are more abstract in nature, such as synaesthetic metaphors (Winter, 2019). Thus, commonalities were sought at this level of abstraction leading to the inclusion of 12 semantic descriptors that appear in Table 1 along with indicative appearances in the timbre and odor semantics literature respectively. This list of semantic descriptors is not exhaustive; however, to the best of the author's knowledge, it represents the first attempt to compile terms that can be equally applied to describe both auditory and olfactory impressions. In addition to this primary criterion, the selection of descriptors was also guided by the goal of providing a parsimonious yet representative set, suitable for empirical investigation. The chosen terms encompass a range of sensory dimensions, including texture (e.g., sharp), size and mass (e.g., full, thin), material (e.g., woody, metallic), temperature (e.g., warm), luminance (e.g., bright), taste (e.g., sweet), and high-level qualities (e.g., rich, ethereal, fresh, complex). The experiment was conducted with Greek native speakers, and as a result, the terms were presented in the Greek language.

**Table 1:** The 12 common semantic descriptors selected for the assessment of auditory and olfactory stimuli.

**Descriptor**	**Timbre semantics references**	**Odor semantics references**
Rich ($\Pi \lambda o\overset{´}{\upsilon }\sigma \iota o$)	Kendall and Carterette, 1991, Zacharakis et al., 2014, Reymore and Huron, 2020	Tekiroǧlu et al., 2014 as cited in Winter, 2019
Thin (Λ*επτó*)	Kendall and Carterette, 1993b, Wallmark, 2019	Parveen et al., 2023, Menini et al., 2022
Full ($\Gamma \varepsilon \mu \overset{´}{\alpha }\tau o$)	von Bismarck, 1974a, Zacharakis and Pastiadis, 2016	Menini et al., 2022
Sweet (Γ*λυκó*)	Fritz et al., 2012, Zacharakis et al., 2014, Reymore and Huron, 2020	Hörberg et al., 2022, Kaeppler and Mueller, 2013
Fresh ($\Phi \rho \overset{´}{\varepsilon }\sigma \kappa o$)	Rodŕıguez et al., 2024, Lynott and Connell, 2009	Jaubert et al., 1995, Rodŕıguez et al., 2024, Hörberg et al., 2022, Lynott and Connell, 2009
Ethereal ($\text{A}\iota \theta \overset{´}{\varepsilon }\rho \iota o$)	Zacharakis et al., 2014, Reymore and Huron, 2020	Jaubert et al., 1995, Zwaardemaker as cited in Boring, 1928
Woody ($\Xi \overset{´}{\upsilon }\lambda \iota \nu o$)	Zacharakis et al., 2014, Reymore and Huron, 2020	Jaubert et al., 1995; Dravnieks et al., 1984; Burlingame et al., 2004
Metallic (Mε*ταλλικó*)	Zacharakis et al., 2014; Reymore and Huron, 2020,	Dravnieks et al., 1984; Burlingame et al., 2004
Warm (Zε*στó*)	Rosi et al., 2023, Rosi et al., 2022, Pratt and Doak, 1976	Dravnieks et al., 1984, Castro et al., 2013
Sharp ($\text{O}\xi \overset{´}{\upsilon }$)	von Bismarck, 1974b, Kendall and Carterette, 1993a, Zacharakis et al., 2014	Jaubert et al., 1995, Dravnieks et al., 1984, Burlingame et al., 2004
Bright (Φ*ωτεινó*)	Fritz et al., 2012, Schubert and Wolfe, 2006, Saitis and Siedenburg, 2020, Saitis and Wallmark, 2024	von Hornbostel, 1931, Cohen, 1934, Lynott and Connell, 2009
Complex ($\Pi o\lambda \overset{´}{\upsilon }\pi \lambda o\kappa o$)	von Bismarck, 1974b, Kendall and Carterette, 1993b, Kendall and Carterette, 1991	Jaubert et al., 1995, Parveen et al., 2023

Each adjective is accompanied by indicative sources from the literature on sound and odor semantics respectively. The original Greek terms appear in parentheses.

### 2.4 Procedure

All participants took part in two experimental blocks, during which they rated sounds and aromas separately using the preselected set of 12 semantic attributes (see Section 2.3). These attributes were presented in the Verbal Attribute Magnitude Estimation (VAME) format (Kendall and Carterette, 1993a,b), where the endpoints of each scale are labeled by the attribute and its negation (e.g., “not rich–very rich”). Participants were allowed to rate each stimulus on as many attributes as they deemed appropriate (including the option to select none), determining their salience using a horizontal slider on a hidden continuous scale ranging from 0 to 100. All sliders were set by default at the negative endpoint (i.e., 0 value). The overall experimental procedure, including instructions, lasted ~30 min for most participants.

The order of blocks was randomized: 12 participants rated aromas first, while the remainder began with sounds. The stimuli presentation order within each block was also randomized. For the sound stimuli, this randomization was managed directly through the Max/MSP GUI, while the aromatic stimuli were manually randomized by the experimenter before each trial. The glass bottles containing the aromas were presented in a row, each labeled with stickers numbered 1 through 12 to track the randomized presentation order for each participant.

## 3 Results

### 3.1 Structural relationships of semantic variables across modalities

**Figure 1:**
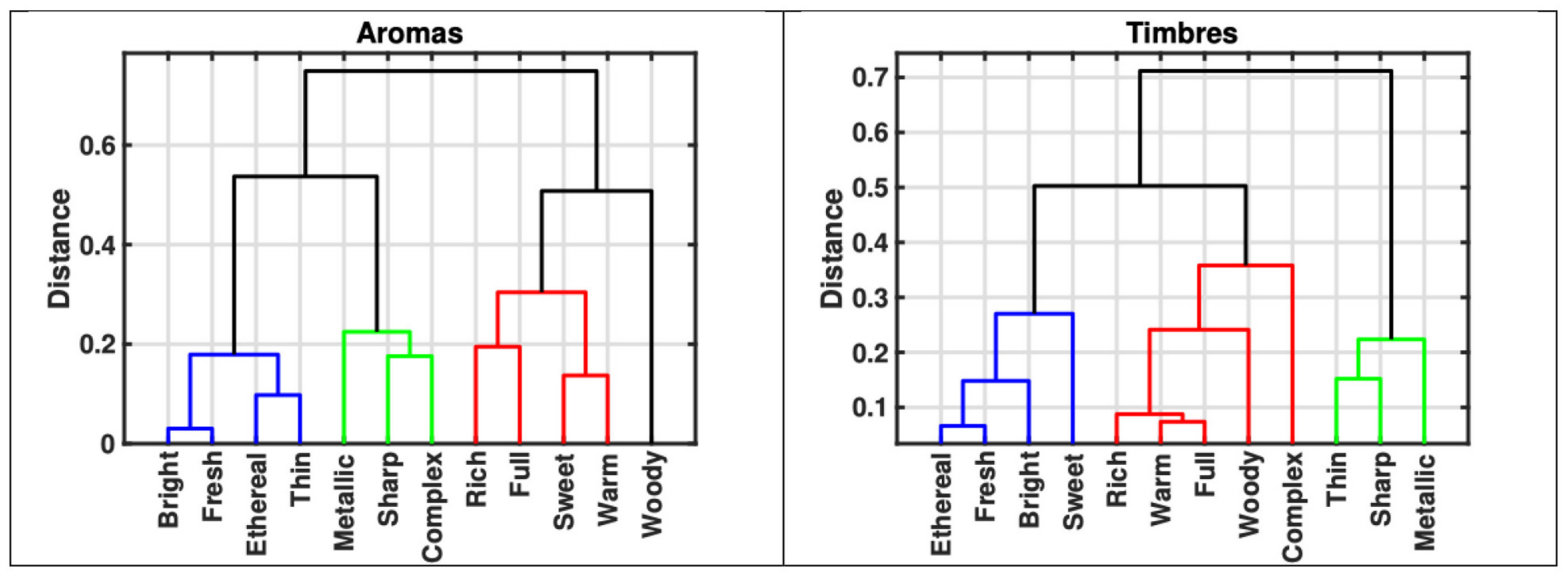
Dendrograms from cluster analysis (complete linkage, cosine similarity) applied to the 12 semantic variables based on mean scores for aromas and sounds. **(Left)** Dendrogram from aroma descriptions. **(Right)** Dendrogram from timbre descriptions. Three major clusters reveal strong conceptual parallels across modalities.

One of the main aims of this study was to evaluate the level of agreement in the organization of semantic variables across the auditory and olfactory modalities. To this end, a Mantel-like permutation test was conducted to assess the overall similarity of the pairwise distances among the 12 semantic variables (calculated by applying cosine similarity on the mean scores) across modalities. The analysis revealed a statistically significant, albeit weak, correlation between the two distance vectors (Spearman's ρ = 0.36, *p* < 0.01), using permutation testing with 10,000 iterations). Following this, two separate cluster analyses were performed (complete linkage, cosine distance metric) to provide a more detailed perspective of the organization of semantic variables within each modality. Figure 1 presents the resulting dendrograms, illustrating the semantic structures emerging from aroma and timbre descriptions, respectively. The structural commonalities between the two dendrograms are more pronounced than what the Mantel test results initially suggested. Notably, both dendrograms reveal three prominent clusters: [*bright, fresh, ethereal*], [*sharp, metallic*], and [*rich, warm, full*]. In contrast, the terms [*thin, sweet, complex*, and *woody*] appear more dispersed, indicating variability in their semantic associations within both modalities. These prominent clusters remain robust even when alternative clustering methods and distance metrics are applied. Overall, these results suggest a meaningful degree of similarity in the semantic structures across the two modalities.

### 3.2 Global semantic distances among timbres and scents

Another goal of the current analysis was to assess the global similarity between the direct distances of sounds and aromas, as derived from Zacharakis (2024), and the indirect distances originating from the scores on the common semantic descriptors collected here. The direct pairwise distances between aromas and timbres, which in this case serve as ground truth, were computed as the median value of the non-zero ratings for each sound–aroma pair, weighted by the relative number of ratings (i.e., no. of ratings ÷ maximum number of ratings observed across all pairs). Indirect distances were estimated using a distance metric on the 12-point vectors.

The Spearman correlation was the preferred distance metric for both indirect distance estimation and the assessment of global similarity, as this combination produced the highest initial global similarity compared to other metrics. The analysis began with the assumption of equal contributions from each semantic scale in forming the indirect distances, resulting in a Spearman correlation of −0.45 (*p* < 0.001) between the two datasets. The negative correlation arose because, in the indirect distance calculation (1 - Spearman correlation), a value of 1 represented the maximum distance, whereas in the direct distance formula, larger values indicated closer associations.

To improve the match, a genetic algorithm (GA) was used to optimize the weights assigned to the semantic scales, testing whether varying the importance of each scale would enhance the correlation. The optimization criterion was to minimize the Spearman correlation between the direct and indirect distances. The choice of GA was motivated by the non-smooth and non-differentiable nature of Spearman correlation, which benefits from GA's exploratory approach in avoiding potential local minima. The GA was configured with a population size of 1,000, a maximum of 100 generations, a mutation rate of 10%, and a crossover fraction of 0.8. An elite count of five ensured the best solutions were preserved, and progress was monitored with results displayed at each iteration. This optimization improved the global distance reaching a Spearman correlation of approximately −0.62, (*p* < 0.001) between the direct and indirect distances.

**Figure 2:**
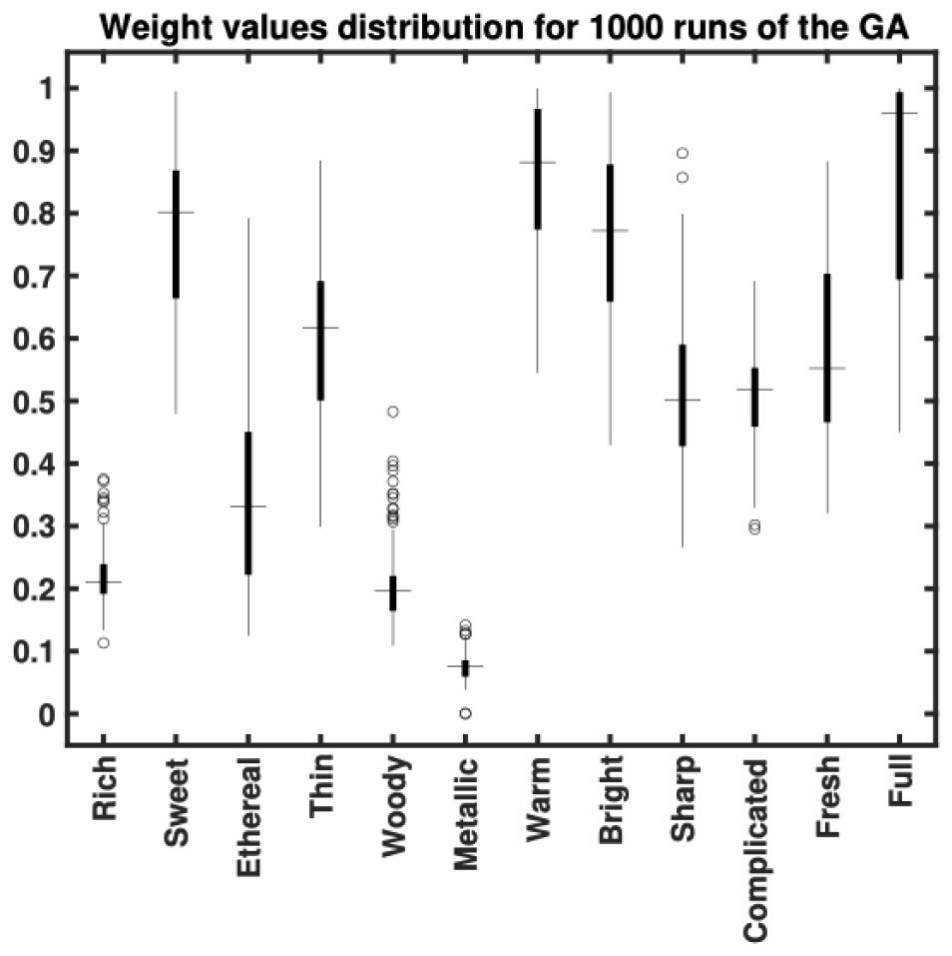
Distribution of weight values assigned for 1,000 runs of the genetic algorithm optimization. This figure indicates that the optimization promotes the importance of some semantic scales (e.g., *sweet, warm, bright* and *full*) over others (e.g., *rich, ethereal, woody* and *metallic*).

Figure 2 shows the distribution of weights generated for 1,000 runs of the GA optimization. It is evident that certain semantic scales are prioritized over others to maximize the fit between the semantically derived distances and the ground truth data. The influence attributed to the descriptors *rich, woody, metallic*, and *ethereal* was suppressed, while *sweet, warm, bright* and *full* were given greater emphasis. *Thin, sharp, complex*, and *fresh* received moderate weightings on average.

### 3.3 Spatial configurations of auditory and olfactory stimuli

Subsequently, one optimized solution was subjected to a non-metric Multidimensional Scaling (MDS) analysis to obtain a common spatial configuration including both timbres and aromas. The dissimilarity matrix for the MDS analysis was created by converting the 12-point semantic vectors into distances, again using the Spearman correlation as the metric.

Table 2 presents the measures-of-fit for 1-, 2-, and 3-dimensional solutions. Kruskal's stress—a measure-of-misfit—exhibits substantial improvement moving from the 1D to 2D solution and a moderate improvement from 2D to 3D. However, the *R*-squared measure of fit slightly decreases from 2D to 3D suggesting that while the 3rd dimension may offer a more precise mapping of the dissimilarities, it does not account for more variance. This could indicate overfitting noise. Therefore, the optimal dimensionality for this dataset was determined to be 2.

**Table 2:** Measures-of-fit and their improvement for different MDS dimensionalities of the semantically derived distances.

**Dimensionality**	**Kruskal's stress**	**Improv**.	***R*-squared**	**Improv**.
1D	0.41	–	0.036	–
2D	0.15	0.26	0.77	0.73
3D	0.09	0.06	0.74	–0.03

**Figure 3:**
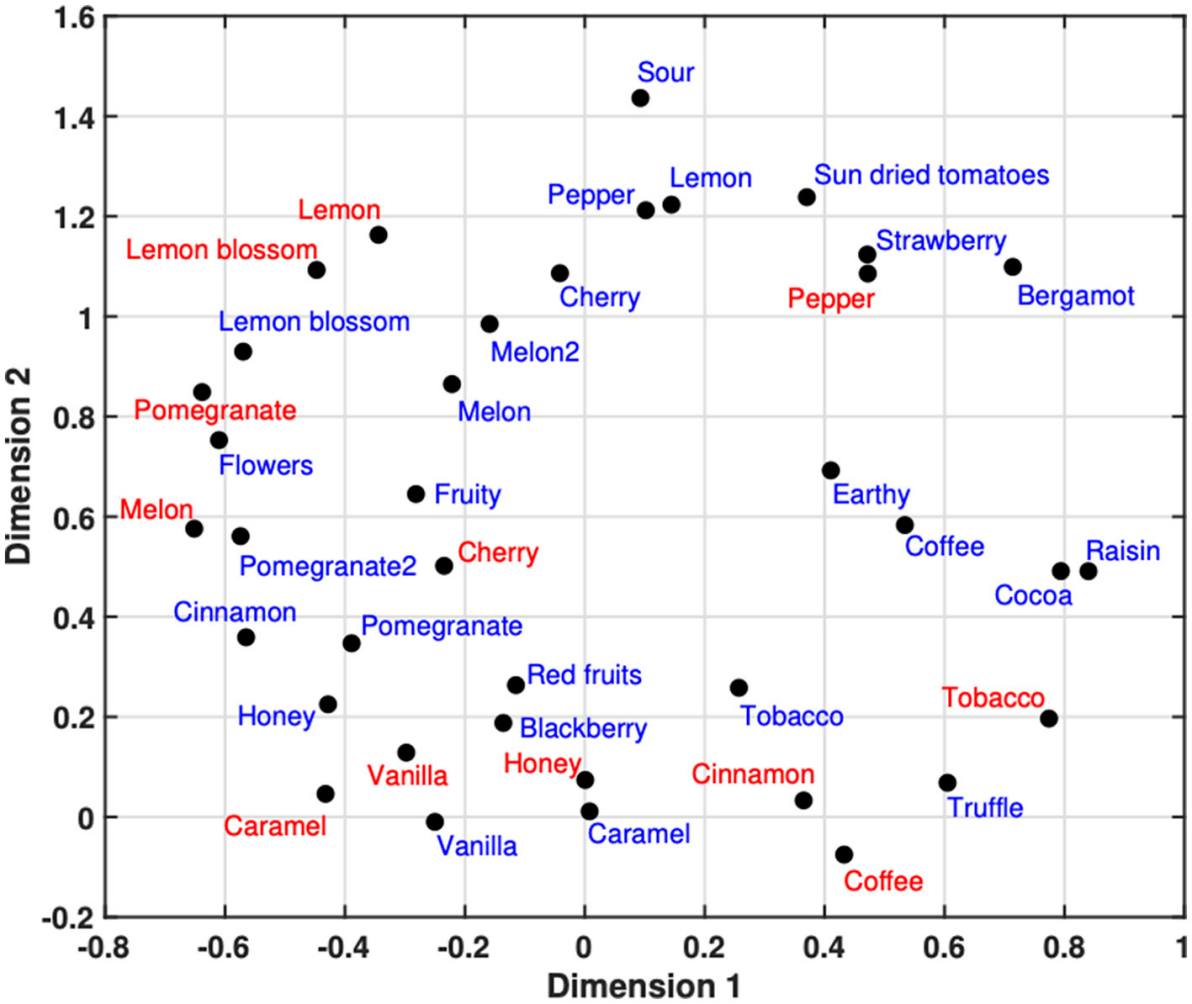
2-dimensional semantic space derived from the conversion of semantic vectors into dissimilarities and subsequent Multidimensional Scaling analysis. Red labels represent the aromatic stimuli and blue labels correspond to the sound stimuli, named after their intended aromatic counterparts. The double versions of sonic pomegranate and melon indicate different realizations of the same aromatic target. The following tentative labeling is suggested based on the semantic profiles of the stimuli (see Supplementary Figures S1, S2). 1st dimension: (+) *complex*/*full* vs. *bright*/*ethereal*/*sweet*/*fresh* (–); 2nd dimension: (+) *thin*/*sharp* vs. *warm*/*full*/*rich* (–).

Figure 3 shows the 2-dimensional MDS space that resulted from the translation of semantic descriptions into dissimilarities. Red tags represent the aromatic stimuli and blue tags the sound stimuli. It should be stressed once more that the sound labels employed derive directly from the names of intended aromatic counterparts as described in Zacharakis (2024).

A visual inspection of the spatial configuration reveals a notable organization of scents. Starting at the top right with *black pepper* and moving clockwise, bitter and spicy odors such as *tobacco, coffee*, and *cinnamon* transition into sweeter aromas clustered at the bottom left, including *caramel, vanilla*, and *honey*. The configuration then rises toward the middle left with fruity scents like *melon, cherry*, and *pomegranate*, before arriving at sour scents at the top left, such as *lemon* and *lemon blossom*.

**Figure 4:**
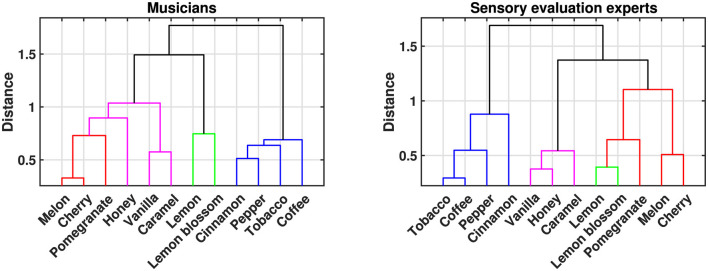
Dendrograms from cluster analysis (complete linkage, distance metric: cosine similarity) applied to the 12 aromatic variables [scores of direct distances with sounds as calculated in Zacharakis (2024)]. **(Left)** Data from musically trained participants (Zacharakis, 2024). **(Right)** Data from experts in sensory evaluation (previously unpublished). The color differentiation of the clusters reflects broad aromatic categories as adopted in this work. Sour: green, fruity: red, sweet: magenta and spices/other: blue.

Interestingly, this organization of scents based on semantic differences seems to align well with the organization produced through cluster analysis (complete linkage, distance metric: cosine similarity) of judgments on direct auditory-olfactory associations. Figure 4 shows this organization in the form of dendrograms. The left one refers to the data collected by a panel of 29 musicians presented in Zacharakis (2024), while the one on the right comes from the ratings by a panel of 25 experts in sensory evaluation (e.g., food scientists, sommeliers, winemakers, etc.) on the same stimuli. The dendrograms between the two groups of participants mainly differ in the clustering of fruity scents (i.e., *melon, cherry* and *pomegranate*). Musicians group them along with sweeter aromas (i.e., *vanilla, caramel* and *honey*) while sensory experts group them with the more sour scents (i.e., *lemon* and *lemon blossom*). The cluster comprising spices and other scents is identical in both groups. This level of organization is also reflected in the MDS space derived from optimized differences in semantic description. The fruity, sour and sweet scents occupy the left half of the space (dimension 1) while the four spices/others occupy the right half. Additionally, fruity aromas are located between sour and sweet ones on the vertical axis (dimension 2), which helps explain the discrepancy in their grouping—either with sweet or sour scents—across the two participant groups.

**Figure 5:**
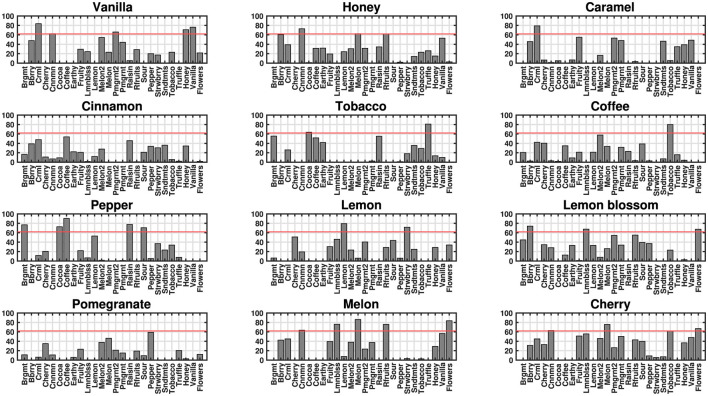
Bar graphs showing the 85th percentiles (vertical axes) of the direct associations between the 12 aromas and each of the 26 sound stimuli (horizontal axes). This high percentile was used to improve the visualization of sparse data, as lower percentiles returned zero values. The statistical significance threshold [indicated by a horizontal red line at 62, (*p* < 0.05)], was determined through a bootstrapping approach with 1,000 random computational simulations of the experimental conditions [Reproduced from Zacharakis (2024), licensed under CC BY-NC 4.0.].

Besides the commonalities in the aromatic organization based on the semantic description of aromas and the one based on direct sound-aroma correspondences, it is also worth focusing on some notable similarities between specific olfactory and auditory stimuli. Figure 5 displays the sounds significantly linked with each scent, as identified by Zacharakis (2024), showing the 85th percentiles of association magnitudes that exceed the chance level (62/100). Statistical significance was determined by employing a bootstrapping simulation replicating the experimental conditions through random sampling. Some of the statistically significant relationships manifest as close pairwise proximities within the auditory-olfactory 2-dimensional semantic space. A few striking examples of shared close cross-modal relationships include the associations of *vanilla scent* with sonic vanilla, honey, and caramel; *tobacco scent* with sonic truffle; *lemon blossom scent* with sonic lemon blossom; *coffee scent* with sonic tobacco; *cherry scent* with sonic melon, etc.

Although no data have been collected on direct perceptual similarities among the sound stimuli yet, there are some indications that semantic descriptions alone were able to represent small differences between sounds. For example, the pairs vanilla-caramel, lemon blossom-flowers, melon-melon2, pomegranate-pomegranate2 originated from variations on common sound synthesis templates and, hence, sound very similar. These similarities are all very accurately represented in the 2D semantic space. The sound stimuli are available in the Supplementary material for interested readers to assess timbral similarities and differences.

Finally, while the 2-dimensional space derives from semantic data, the labeling of its MDS-generated dimensions remains open to interpretation. Along dimension 1, stimuli at the positive end are associated with qualities like *complex* and *full*, including sonic coffee, cocoa, raisin, and tobacco aroma. The negative end, which includes aromas such as melon and pomegranate as well as sonic flowers, cinnamon, and pomegranate2, generally reflects higher scores in *sweet, ethereal, fresh*, and *bright*. For dimension 2, the positive end aligns with *thin* and *sharp*, with scents like lemon, lemon blossom, and pepper and timbres such as sour, lemon, bergamot, strawberry and pepper receiving strong ratings on these attributes. In the negative end, timbres like vanilla, caramel, and truffle rate highly in *full* and *rich*, while corresponding scents (honey, cinnamon, coffee, caramel, and vanilla) display a mix of *warm* and *full* with *sweet*. Potential explanations for this discrepancy (also evident in the left dendrogram of Figure 1) will be offered in the following discussion. Radar plots presenting the semantic profiles for the aromatic and timbral stimuli used in this experiment are provided in the Supplementary material.

## 4 Discussion

This paper presented a follow up study of recent research on cross-modal correspondences between aromatic oils and synthetic timbres (Zacharakis, 2024). The current work aimed to examine the extent to which previously identified relationships could be attributed to some common underlying semantic qualities. In other words, whether semantic mediation might account for some of the identified correspondences between olfaction and audition. The ineffability of olfaction –and to a lesser degree audition– together with a common reliance on source-based descriptors, poses a unique challenge. Given the lack of common sources across these modalities, it became essential to consider abstract descriptive terms applicable to both scent and timbre. As highlighted in the introduction, this approach is not uncommon in timbre semantics research (Wallmark and Kendall, 2018; Saitis and Weinzierl, 2019).

Following a literature review, twelve semantic variables were selected. Both the choice of specific terms and the balance of concepts may be open to debate. For instance, descriptors related to fullness (i.e., rich, full, ethereal, thin) make up one-third of the variables, while visually derived metaphors are limited to a single term (i.e., brightness). Nonetheless, as the first study to attempt a direct semantic comparison between scent and timbre, a certain degree of exploratory freedom seems justified.

The structure of the semantic variables for both modalities was examined through cluster analysis. This approach revealed three primary clusters with notable commonalities across the two modalities (Figure 1). This finding contrasts slightly with the weak Spearman's ρ = 0.36, *p* < 0.05 observed when assessing the overall similarity between semantic variables across modalities. Nevertheless, the presence of hierarchical similarities suggests that a higher-level semantic grouping (clustering) is more robust across modalities than the finer pairwise distances between variables would imply. In other words, while exact correlations between the semantic variables may be weak, participants still seem to understand the broader semantic categories in a similar fashion across senses.

Turning to the specific concepts that emerged from the cluster analysis, the first cluster (in blue) appears to align with attributes of brightness, freshness, and ethereality; the second cluster (in red) centers around descriptors such as fullness, richness, and warmth; and the third cluster groups together qualities like sharpness and metallic character. This structure is reminiscent of the salient semantic dimensions of luminance, texture and mass previously reported for timbral semantics (Zacharakis et al., 2014; Zacharakis and Pastiadis, 2016). Another similarity concerns the term woody, which appears less associated with the other descriptors, particularly in aroma descriptions, but also to a degree in timbre. Although woody loosely aligns with the full/rich cluster, it shows relative independence across both modalities. In contrast, thin alternates between the bright/fresh cluster for aromas and the sharp/metallic cluster for timbres, which, however, is not conceptually unexpected. A similar pattern appears with complex, which tends to be more independent within timbre descriptions (loosely associated with full/rich) but aligns with the sharp/metallic cluster in aroma semantics. Finally, sweet also alternates between the bright/fresh cluster in timbre semantics, and the full/rich cluster in aromas semantics. Future work should examine whether these differences reflect systematic differences in how these descriptive terms are used to convey timbral and aromatic qualities, or whether they are driven by specific characteristics of the present stimulus set.

Following the identification of commonalities in the semantic organization present in aroma and timbre semantics this work proceeded to compare direct associations between timbres and scents (converted into distances) from Zacharakis (2024) with distances derived from ratings on the twelve semantic scales. Converting ratings on semantic variables into perceptual distances and MDS spaces has been proven meaningful in past timbre research (Zacharakis and Pastiadis, 2016). Even without making any assumptions regarding the weight attributed to the separate semantic descriptors, a moderate but significant Spearman correlation was obtained between the two distance vectors. However, a genetic algorithm (GA) optimization of the Spearman correlation between them yielded a stronger value, reaching 0.62 *p* < 0.001. This level of correlation is remarkable when considering the inevitable information loss involved in the processes of cross-modal associations, semantic descriptions and distance estimations.

The distribution of weights from multiple runs of the optimization algorithm indicated that the source property descriptors *metallic* and *woody*, along with *rich* were suppressed to achieve a better fit of the semantically derived distances to the direct ones. Conversely, descriptors such as *full, warm* (representing the red cluster), *bright* (representing the blue cluster) and *sweet* were the most strongly weighted on average (albeit with wide interquartile ranges). Representatives of the green cluster such as *sharp, thin* or *complex* received moderate weightings on average.

It is reasonable to assume that semantic descriptors receiving stronger weights may play a more critical role in the cross-modal relationships between scents and sounds. In contrast, the suppression of some descriptors' weights could imply that those attributes are less relevant. However, it should again be acknowledged that these weightings could simply reflect the particular characteristics of the aromatic and sonic stimuli employed in this study. It is possible that the current stimulus set emphasizes certain semantic attributes over others, thus shaping the observed semantic prominence. Therefore, while these weightings provide valuable insights into shared semantic salience between aromas and timbres, further work on cross-modal correspondences between audition and olfaction is necessary before reaching robust generalizations.

Beyond the global similarities, examining the specific positioning of stimuli within the MDS-generated semantic space offers further insights into how semantic properties can account for direct cross-modal correspondences. The spatial organization of scents in this 2D space (Figure 3) is not only conceptually coherent but also resembles the arrangement observed in direct aroma-timbre correspondences obtained separately from musicians and sensory evaluation experts (Figure 4). This suggests that both the semantic descriptions of aromas and the aroma-timbre associations reveal comparable relationships among this group of scents.

The fact that the sound stimuli were predominantly synthetic, presents limitations for conceptual evaluation of whether timbres are meaningfully positioned in the 2D space. However, their labels, which reflect the intended aromatic target of sound synthesis, indicate that the placement of sound stimuli is also non-arbitrary. An attempt to label the semantic dimensions was made by consulting the semantic profiles (see Supplementary Figures S1, S2) of stimuli positioned at the extremes of each axis. This analysis suggests that dimension 1 represents a spectrum from *complex/full* to *bright/ethereal/sweet/fresh*, while dimension 2 spans from *thin*/*sharp* to *warm/full/rich*.

A final piece of evidence advocating for semantic mediation of timbre-aroma associations is added by the fact that many of the statistically significant cross-modal relationships depicted at Figure 5 are also manifested as close proximities in the semantic space. This is yet another indication that semantic description was able to capture direct cross-modal relationships.

Taken together, these findings suggest that cross-modal associations between auditory and olfactory stimuli may, to some extent, be underpinned by a common semantic substrate, where cross-modal differences and similarities arise through assessments on a number of shared latent qualities. In a similar fashion, perceptual timbre spaces reflecting relationships unmediated by linguistic expression have shown substantial commonalities with semantic timbre spaces (Zacharakis et al., 2015). Although the comparison in this case was unimodal, the premise remains parallel: whether evaluating unimodal or cross-modal similarity, at least part of the judgment appears to be reached by integrating underlying semantic qualities.

This study opens up several potential avenues for future investigation. Since the aromatic substances used in this study were aromatically complex and comprised of mixtures of chemical compounds, it would be meaningful to take a step back and explore odors derived from a single chemical compound. This approach would facilitate the identification of specific semantic labels and allow for a more direct linkage between acoustic characteristics and isolated chemical substances. In addition, although out-of-context identification of odor sources is notoriously challenging (e.g., Yeshurun and Sobel, 2010), preliminary tests of odor and sound source recognition could prove valuable for uncovering whether source familiarity influences the mechanisms shaping auditory-olfactory correspondences. The exploration of emotional mediation alongside semantic influence constitutes another research path worth pursuing, particularly for unfamiliar aromatic stimuli (Spence, 2020b). This becomes even more intriguing in light of recent findings by Reymore and Lindsey (2025), which suggest that semantic mediation plays a more prominent role than emotional mediation in timbre-color correspondences. Given the evidence indicating that emotional mediation becomes more relevant as the complexity of the stimuli increases (Spence, 2020a; Di Stefano et al., 2024), could it be that olfaction carries more emotional significance than vision in its correspondence with audition even for simple stimuli? Finally, constructing shared perceptual timbre-odor spaces from non-lexical pairwise dissimilarities can provide a ground truth for gaining deeper insights into the perceptual relevance of descriptive terms within the realm of sound-aroma correspondences.

Overall, the current findings can act as a starting point for developing a more nuanced and generalizable semantic framework for the description of sonic and aromatic impressions. Such a framework will not only serve to advance our understanding of cross-modal correspondences but could also find its way out of the lab and into scientifically oriented applications in marketing (Spence et al., 2021b; Spence and Keller, 2024; Velasco and Spence, 2024) and real-world multisensory experiences (Spence, 2021).

## Data Availability

The original contributions presented in the study are included in the article/Supplementary material, further inquiries can be directed to the corresponding author.

## Footnotes

1 It should be noted that while interpretations of “Semantics” can vary across disciplines and scholars, this work adopts a broad perspective, wherein meaning emerges when a percept evokes something beyond itself (Patel, 2008).
