# Correction: The poetry of senses: exploring semantic mediation in timbre-aroma correspondences

**DOI:** 10.3389/fpsyg.2025.1744050

**Published:** 2025-12-16

**Authors:** Asterios Zacharakis

**Affiliations:** School of Music Studies, Aristotle University of Thessaloniki, Thessaloniki, Greece

**Keywords:** semantic mediation, timbre, aroma, cross-modal correspondences, audition, olfaction

There was a mistake in Figure 1 as published. The labels of three variables (Sweet, Thin, and Ethereal) were interchanged and are corrected as follows: ethereal → thin, thin → sweet, sweet → ethereal. The two corrected subfigures of corrected Figure 1 appear below.

**Figure 1:**
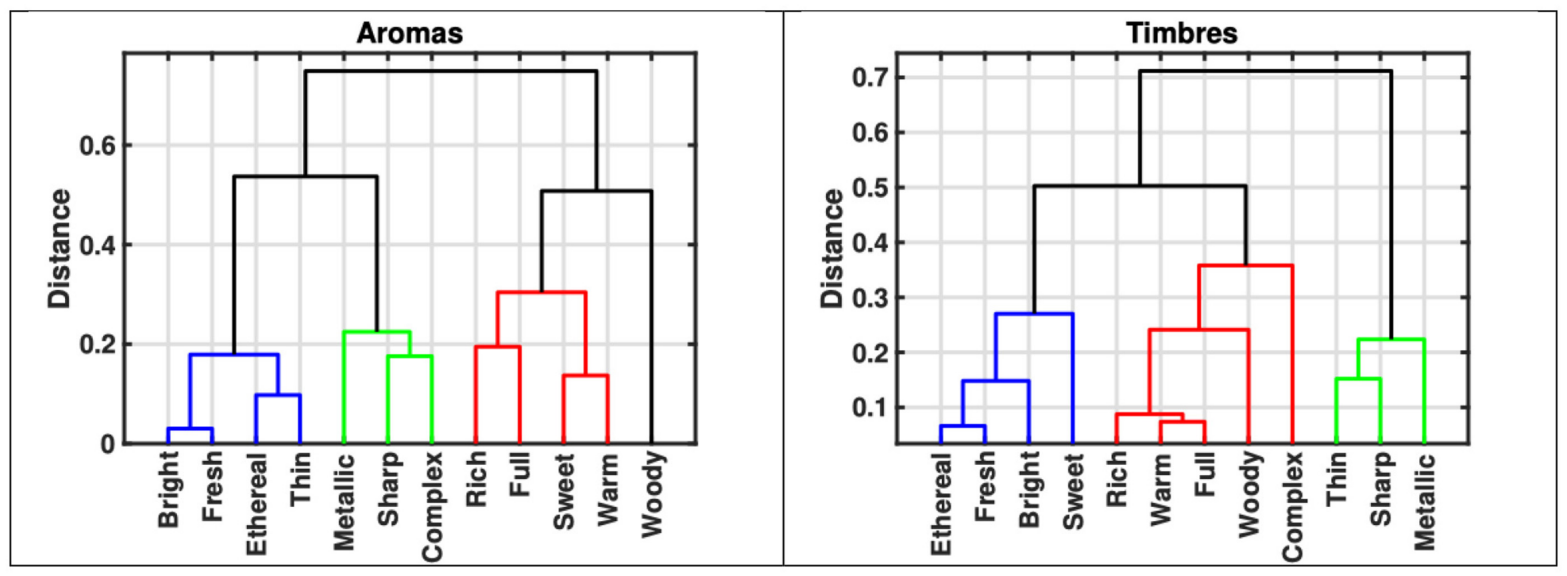
Dendrograms from cluster analysis (complete linkage, cosine similarity) applied to the 12 semantic variables based on mean scores for aromas and sounds. **(Left)** Dendrogram from aroma descriptions. **(Right)** Dendrogram from timbre descriptions. Three major clusters reveal strong conceptual parallels across modalities.

There was a mistake in Figure 2 as published. The labels of three variables (Sweet, Thin, and Ethereal) were interchanged and are corrected as follows: ethereal → thin, thin → sweet, sweet → ethereal.

There was a mistake in the caption of Figure 2 as published. The labels of three of the variables (i.e., Sweet, Thin and Ethereal) were originally interchanged and are corrected as follows: ethereal → thin, thin → sweet, sweet → ethereal. The corrected Figure 2 and its caption appear below.

**Figure 2:**
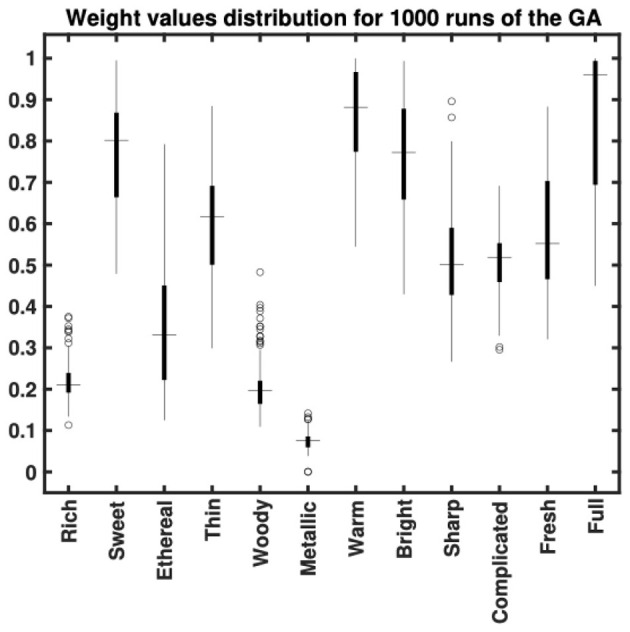
Distribution of weight values assigned for 1,000 runs of the genetic algorithm optimization. This figure indicates that the optimization promotes the importance of some semantic scales (e.g., *sweet, warm, bright* and *full*) over others (e.g., *rich, ethereal, woody* and *metallic*).

There was a mistake in the caption of Figure 3 as published due to incorrect variable labelling. The corrected caption of Figure 3 appears below.

Figure 3. 2-dimensional semantic space derived from the conversion of semantic vectors into dissimilarities and subsequent Multidimensional Scaling analysis. Red labels represent the aromatic stimuli and blue labels correspond to the sound stimuli, named after their intended aromatic counterparts. The double versions of sonic pomegranate and melon indicate different realizations of the same aromatic target. The following tentative labeling is suggested based on the semantic profiles of the stimuli (see Supplementary Figures S1, S2). 1st dimension: (+) *complex*/*full* vs. *bright*/*ethereal*/*sweet*/*fresh* (–); 2nd dimension: (+) *thin*/*sharp* vs. *warm*/*full*/*rich* (–).

The second descriptor of Table 1: Thin/delicate, was corrected to simply Thin. The caption was modified to reflect this change. The corrected caption of Table 1 appears below.

“Each adjective is accompanied by indicative sources from the literature on sound and odor semantics respectively. The original Greek terms appear in parentheses.”

In the abstract, a semantic variable labelling error was made. This has been corrected to read:

“The analysis of semantic variables identified a largely consistent organization for both modalities, condensing into three prominent clusters: [*bright, fresh, ethereal*], [*sharp, metallic*], and [*full, rich, warm*].”

The labels of three variables (Sweet, Thin, and Ethereal) were interchanged.

A correction has been made to the section 3. **Results**, *3.1 Structural relationships of semantic variables across modalities*, Paragraph 1:

“Notably, both dendrograms reveal three prominent clusters: [*bright, fresh, ethereal*], [*sharp, metallic*], and [*rich, warm, full*]. In contrast, the terms [*thin, sweet, complex*, and *woody*] appear more dispersed, indicating variability in their semantic associations within both modalities.”

A correction has been made to the section 3. **Results**, *3.2 Global semantic distances among timbres and scents*, Paragraph 4:

“The influence attributed to the descriptors *rich, woody, metallic*, and *ethereal* was suppressed, while *sweet, warm, bright* and *full* were given greater emphasis. *Thin, sharp, complex*, and *fresh* received moderate weightings on average.”

A correction has been made to the section 3. **Results**, *3.3 Spatial configurations of auditory and olfactory stimuli*, Paragraph 9:

“The negative end, which includes aromas such as melon and pomegranate as well as sonic flowers, cinnamon, and pomegranate2, generally reflects higher scores in *sweet, ethereal, fresh*, and *bright*. For dimension 2, the positive end aligns with *thin* and *sharp*, with scents like lemon, lemon blossom, and pepper and timbres such as sour, lemon, bergamot, strawberry and pepper receiving strong ratings on these attributes. In the negative end, timbres like vanilla, caramel, and truffle rate highly in *full* and *rich*, while corresponding scents (honey, cinnamon, coffee, caramel, and vanilla) display a mix of *warm* and *full* with *sweet*.”

A correction has been made to the section **Discussion**, Paragraph 2:

“…(i.e., rich, full, ethereal, thin)…”

A correction has been made to the section **Discussion**, Paragraph 4:

“Turning to the specific concepts that emerged from the cluster analysis, the first cluster (in blue) appears to align with attributes of brightness, freshness, and ethereality; … In contrast, thin alternates between the bright/fresh cluster for aromas and the sharp/metallic cluster for timbres, which, however, is not conceptually unexpected. A similar pattern appears with complex, which tends to be more independent within timbre descriptions (loosely associated with full/rich) but aligns with the sharp/metallic cluster in aroma semantics. Finally, sweet also alternates between the bright/fresh cluster in timbre semantics, and the full/rich cluster in aromas semantics. Future work should examine whether these differences reflect systematic differences in how these descriptive terms are used to convey timbral and aromatic qualities, or whether they are driven by specific characteristics of the present stimulus set.”

A correction has been made to the section **Discussion**, Paragraph 5. Paragraph 5 was removed.

A correction has been made to the section **Discussion**, Paragraph 7:

“Conversely, descriptors such as *full, warm* (representing the red cluster), *bright* (representing the blue cluster) and *sweet* were the most strongly weighted on average (albeit with wide interquartile ranges). Representatives of the green cluster such as *sharp, thin* or *complex* received moderate weightings on average.”

A correction has been made to the section **Discussion**, Paragraph 8:

“However, it should again be acknowledged that these weightings could simply reflect the particular characteristics of the aromatic and sonic stimuli employed in this study.”

A correction has been made to the section **Discussion**, Paragraph 10:

“This analysis suggests that dimension 1 represents a spectrum from *complex/full* to *bright/ethereal/sweet/fresh*, while dimension 2 spans from *thin*/*sharp* to *warm/full/rich*.”

The figure and raw data files were erroneously published with the original version of this paper. The supplementary files have now been replaced.

The original version of this article has been updated.

