# Supplementary material for: The poetry of senses: exploring semantic mediation in timbre-aroma correspondences
Source: Front Psychol. 2025 Feb 25;16:1520046. doi: 10.3389/fpsyg.2025.1520046 (PMC11894736; doi:10.3389/fpsyg.2025.1520046)
Supplement: Supplementary file 1 [file Data_Sheet_1.zip › Supplementary material_poetry_of_senses_2025/Figures/Supplemetal figures captions.rtf]

Caption FigS1: Radar plots showing the semantic profiles of the 26 timbres, estimated as the weighted median values of non-zero ratings for each sound. Weights are calculated as the number of non-zero ratings on each scale divided by the maximum number of ratings observed across all scales and sounds. The sounds are named after their intended aromatic counterparts (see Zacharakis, 2024).Caption FigS2: Radar plots showing the semantic profiles of the 12 aromas, estimated as the weighted median values of non-zero ratings for each aroma. Weights are calculated as the number of non-zero ratings on each scale divided by the maximum number of ratings observed across all scales and aromas. 
