# Supplementary figures and images for: The poetry of senses: exploring semantic mediation in timbre-aroma correspondences
Source: Front Psychol. 2025 Feb 25;16:1520046. doi: 10.3389/fpsyg.2025.1520046 (PMC11894736; doi:10.3389/fpsyg.2025.1520046)

### Vanilla

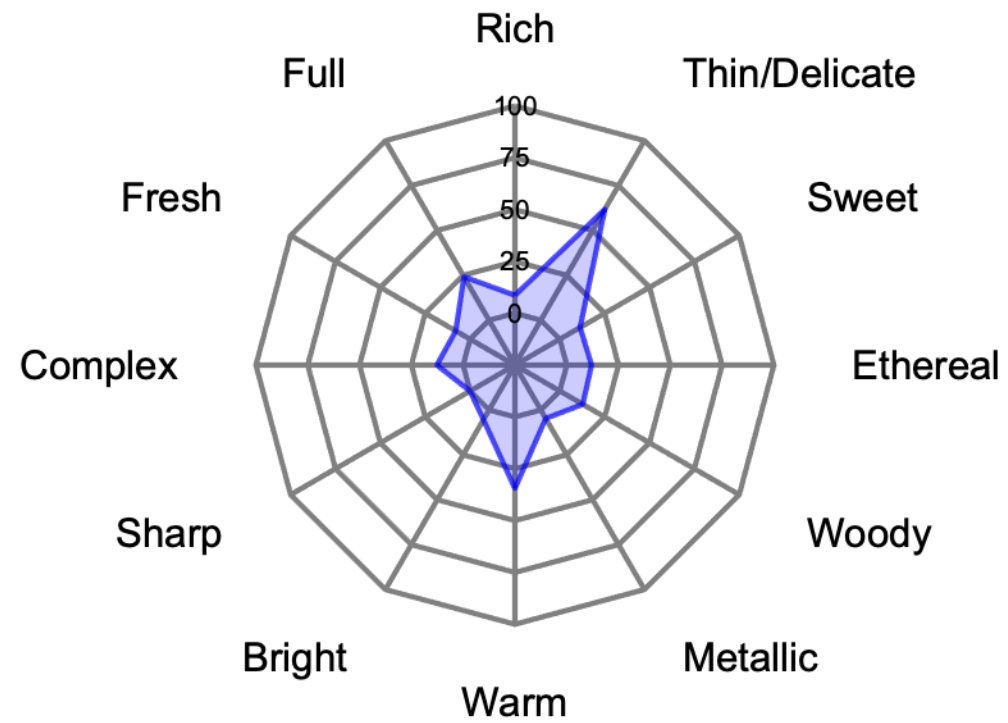

### Honey

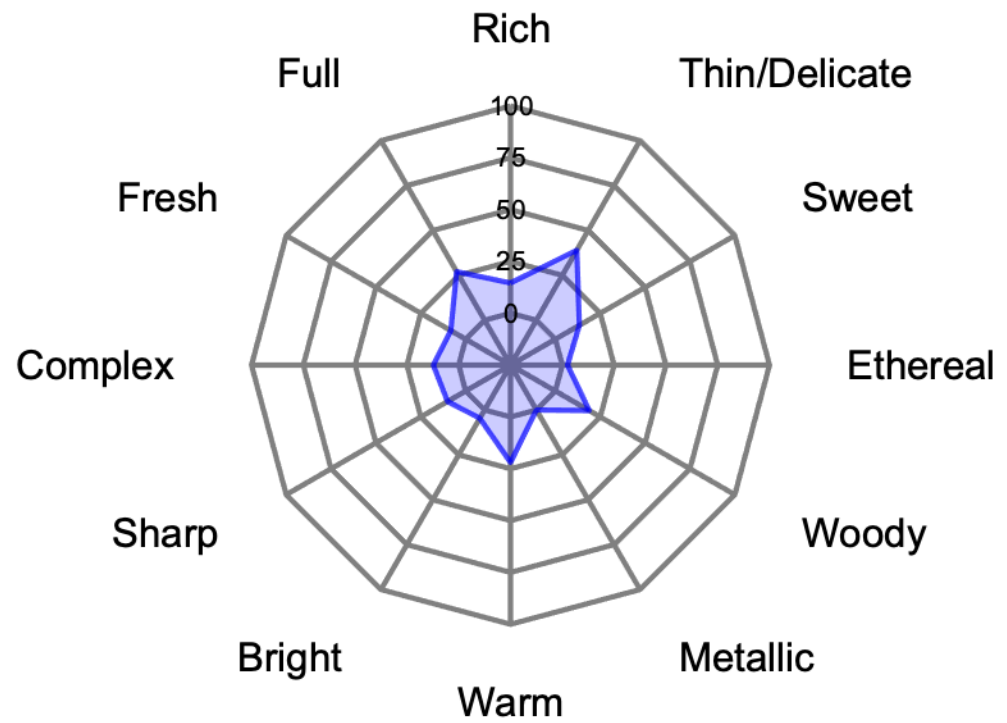

### Caramel

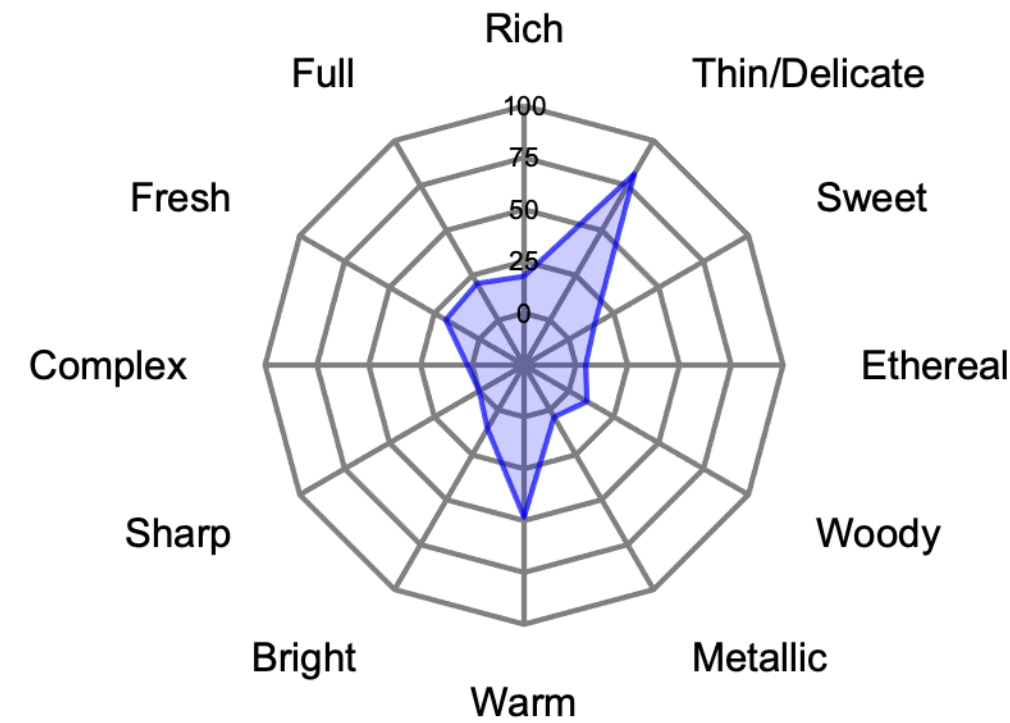

### Cinnamon

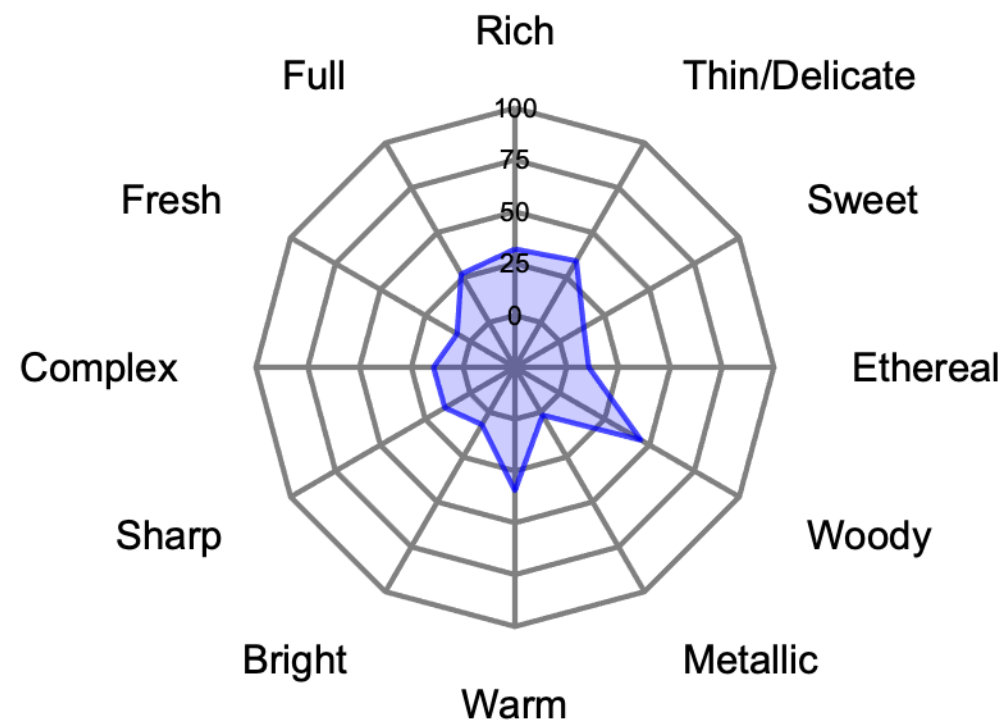

### Tobacco

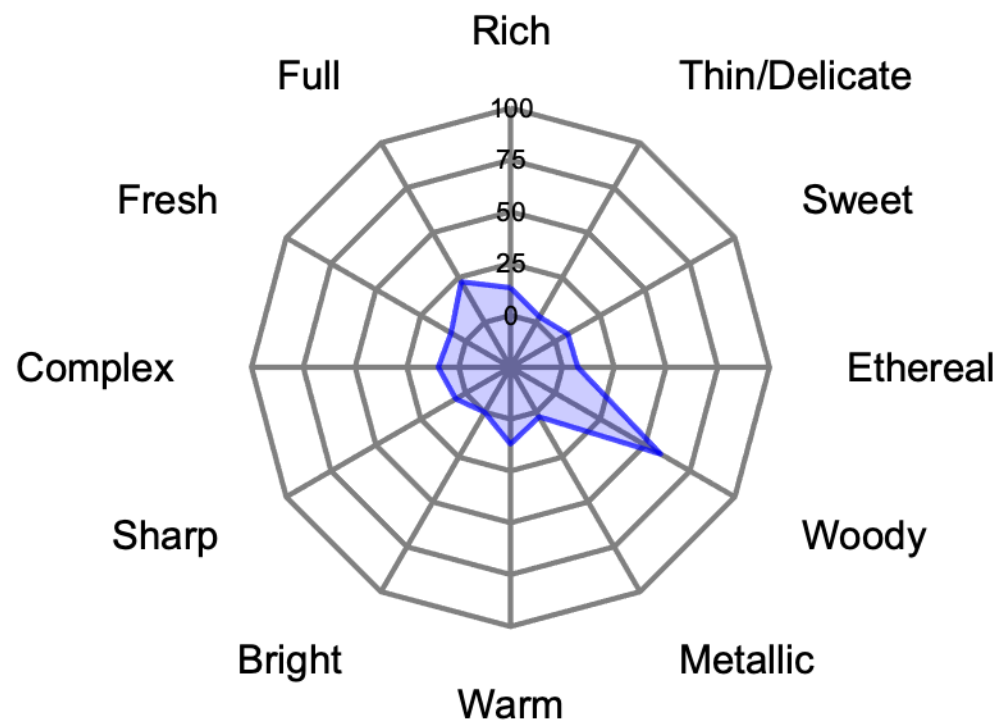

### Coffee

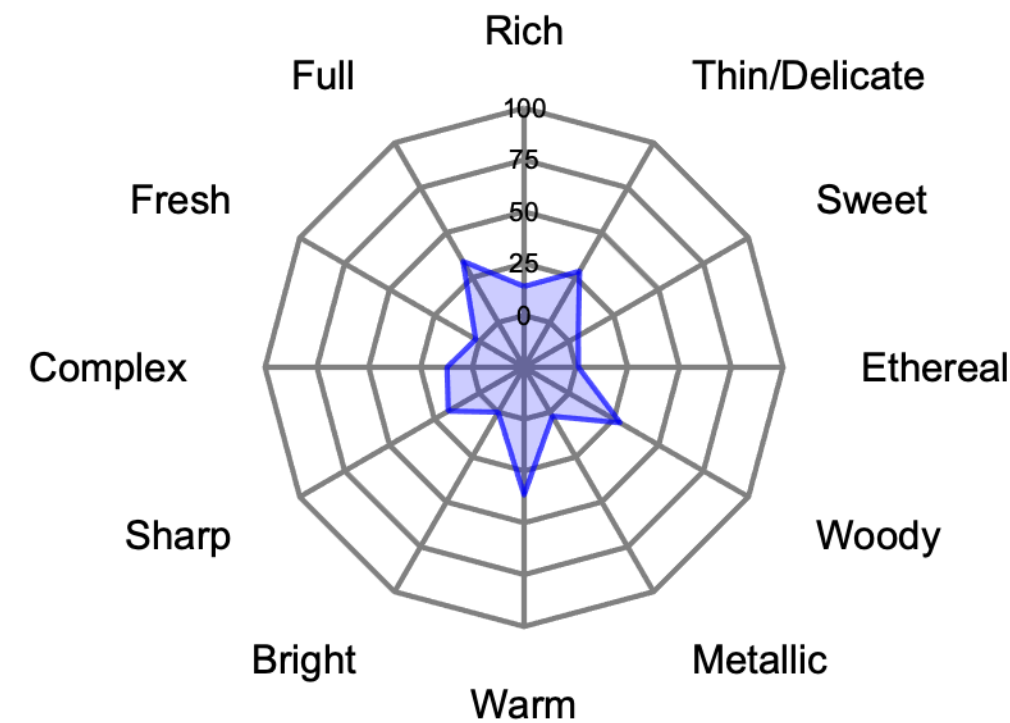

Supplement: Supplementary file 1 [file Data_Sheet_1.zip › Supplementary material_poetry_of_senses_2025/Figures/FigS2a.pdf]

**Pepper**

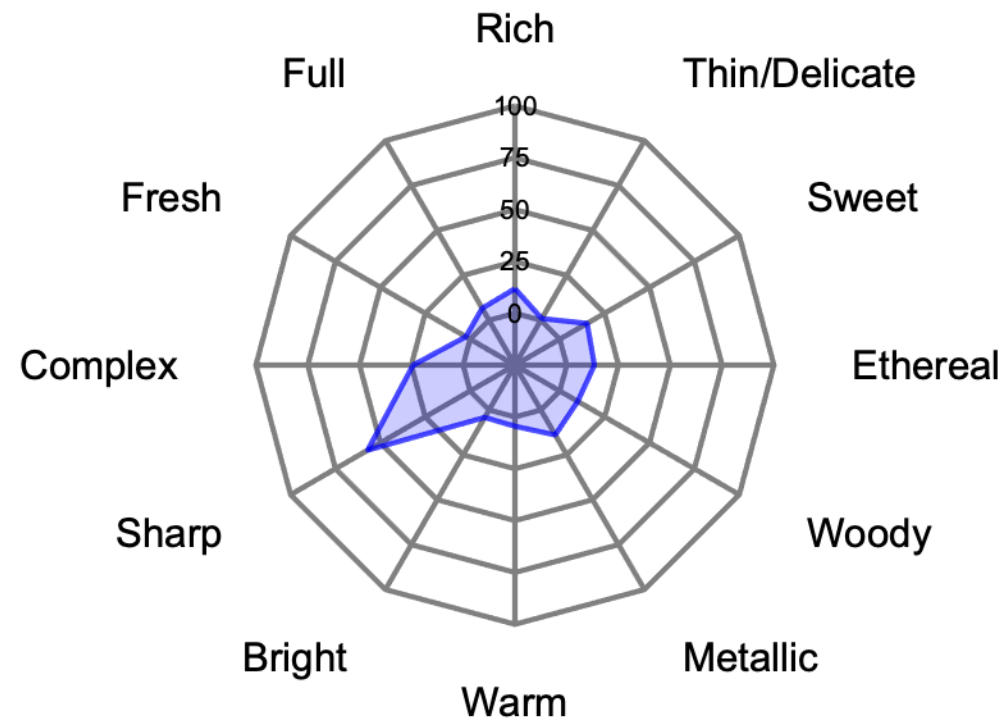

**Lemon**

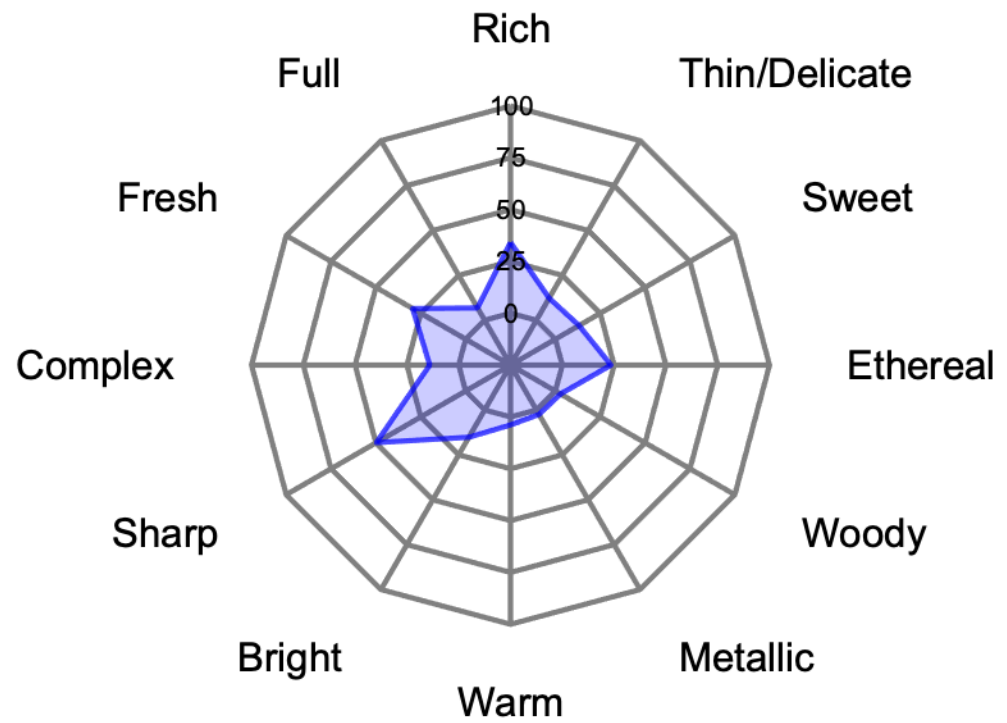

**Lemonblossom**

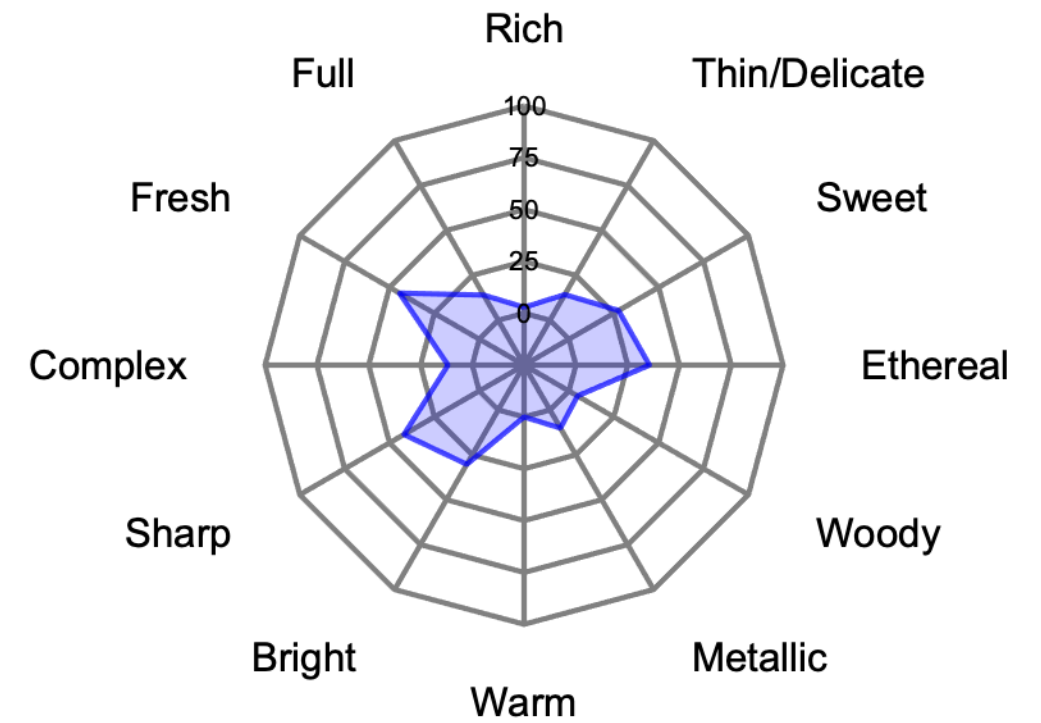

**Pomegranate**

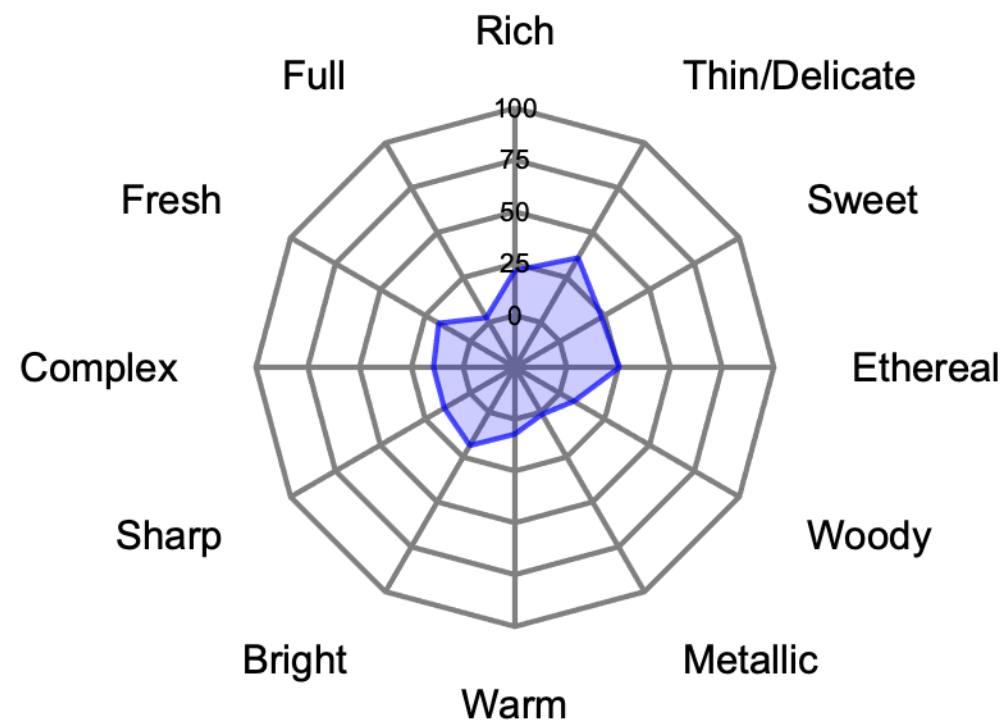

**Melon**

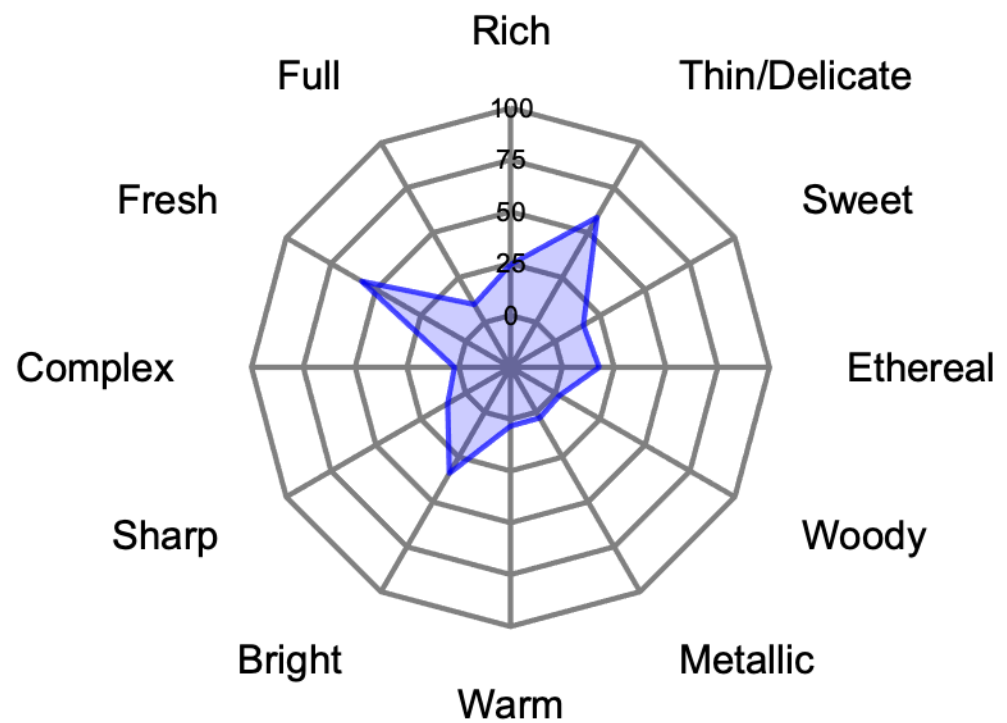

**Cherry**

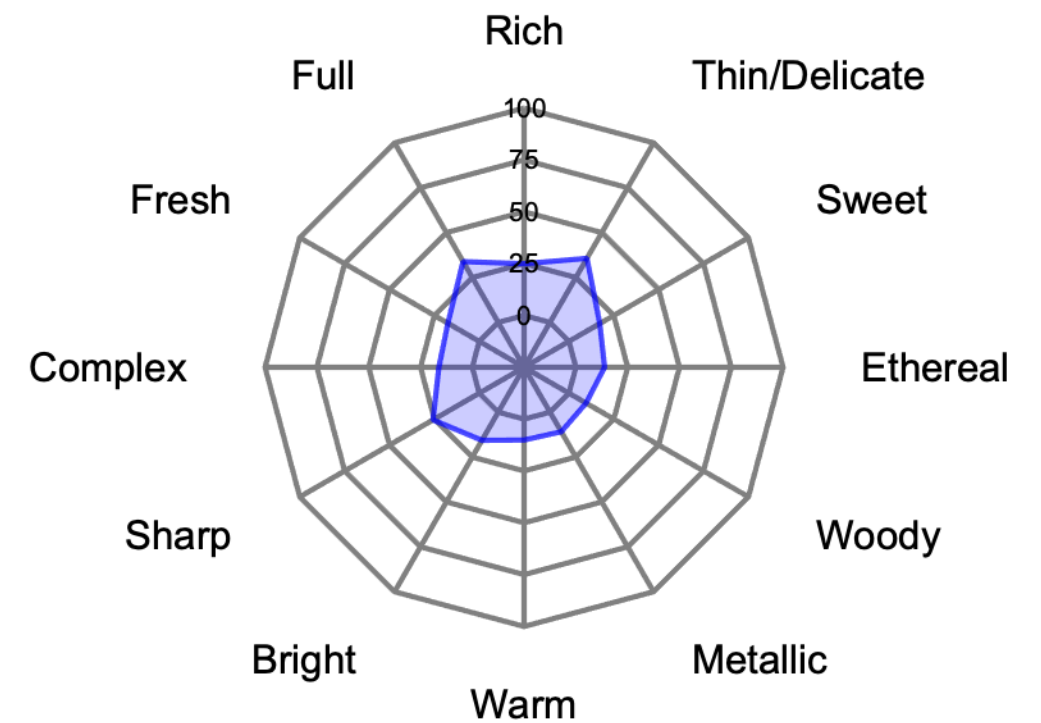

Supplement: Supplementary file 1 [file Data_Sheet_1.zip › Supplementary material_poetry_of_senses_2025/Figures/FigS2b.pdf]

**Pepper**

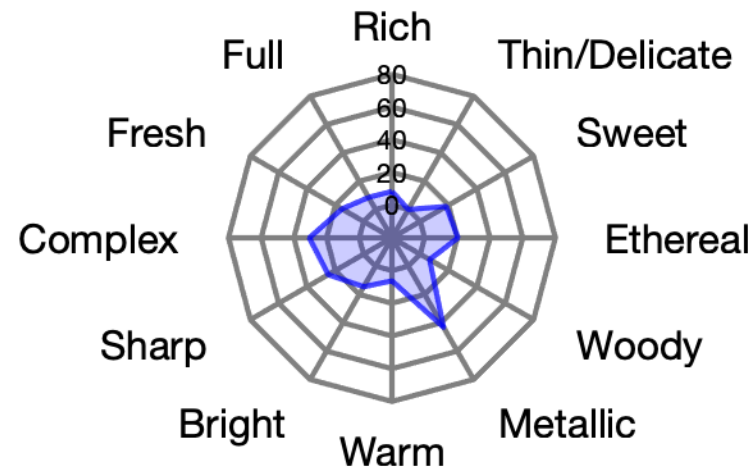

**Strawberry**

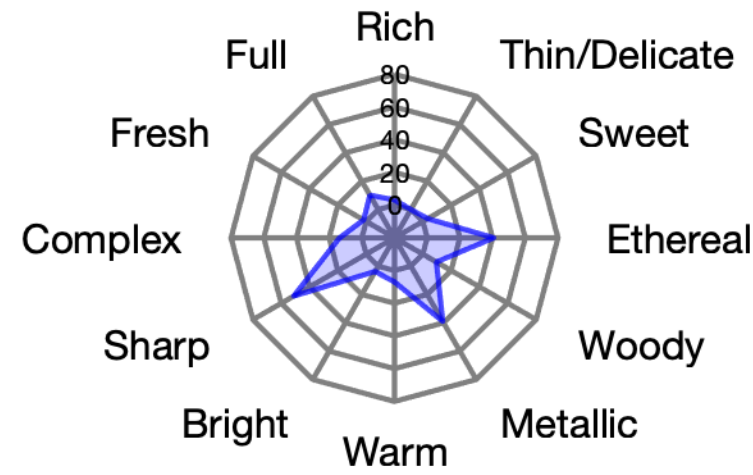

**Sun dried tomatoes**

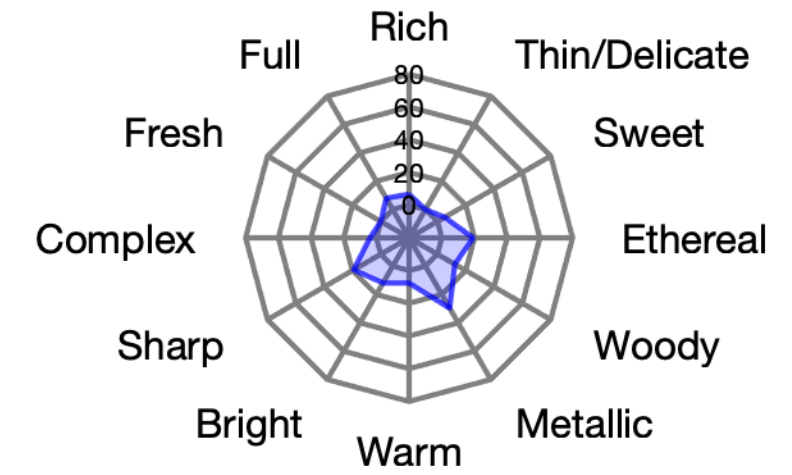

**Tobacco**

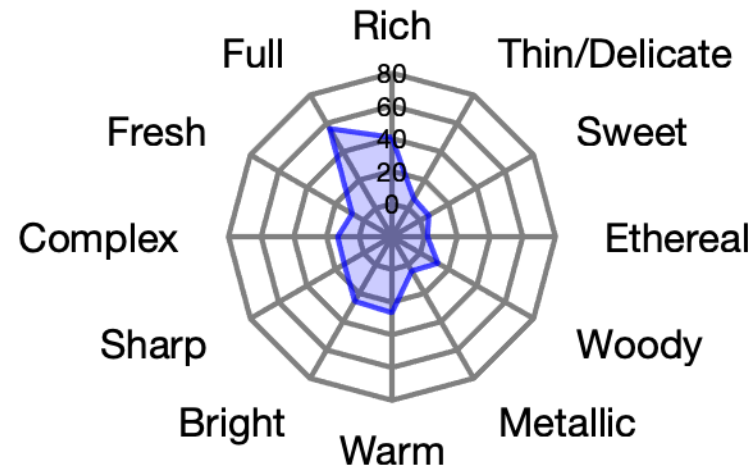

**Truffle**

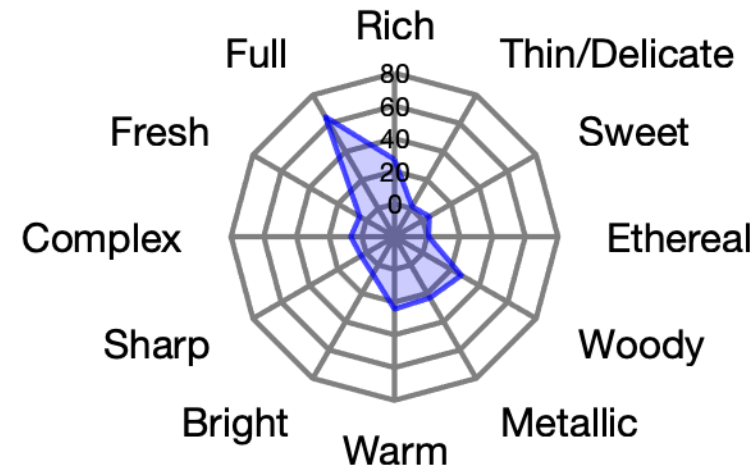

**Honey**

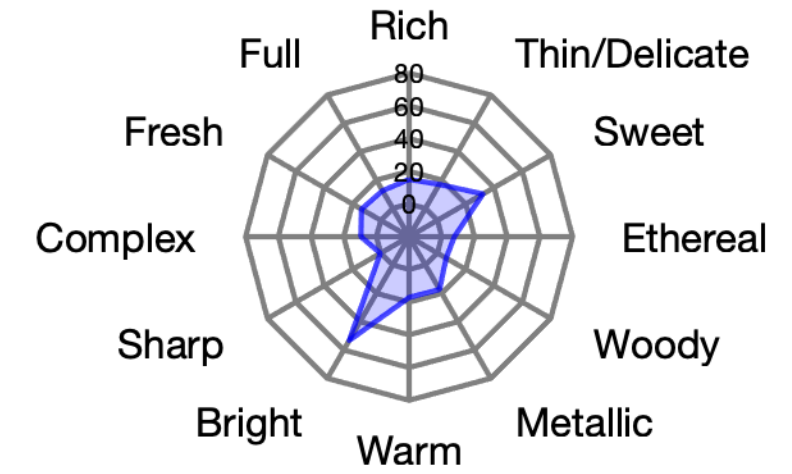

**Vanilla**

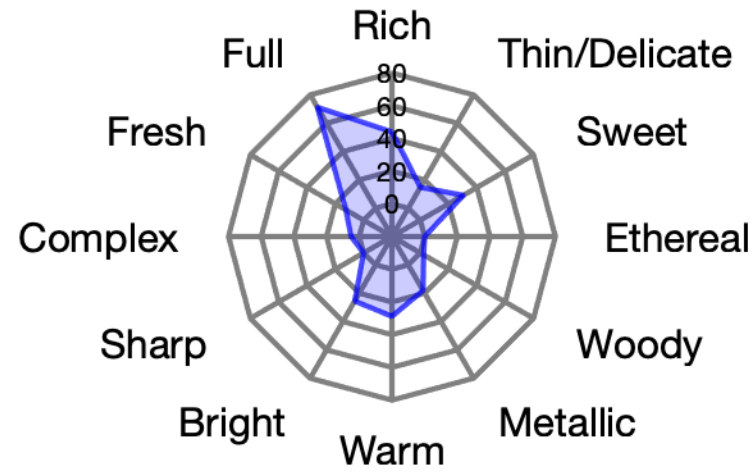

**Flowers**

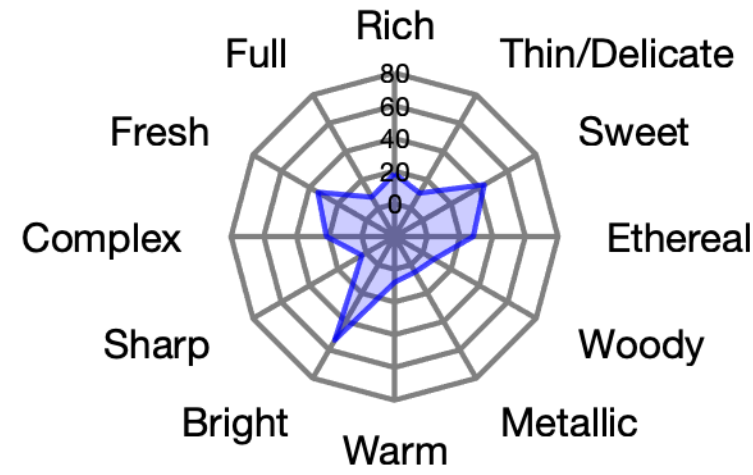

Supplement: Supplementary file 1 [file Data_Sheet_1.zip › Supplementary material_poetry_of_senses_2025/Figures/FigS1c.pdf]

**Bergamot**

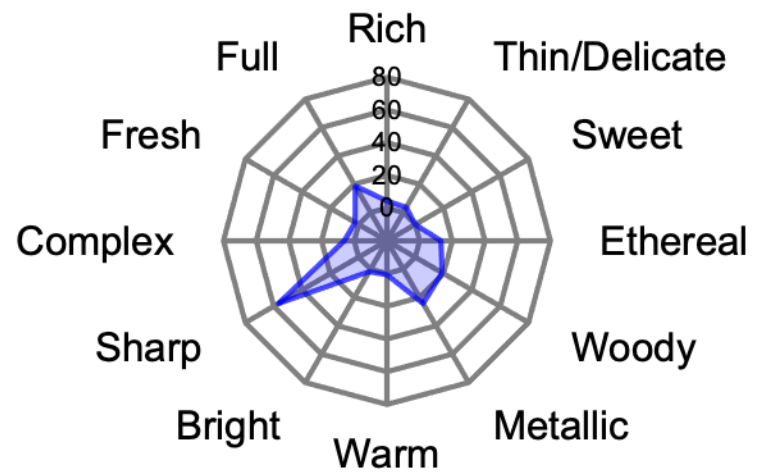

**Blackberry**

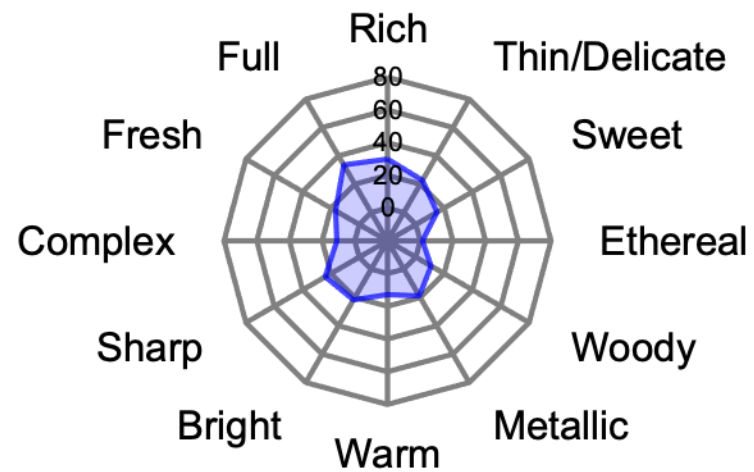

**Caramel**

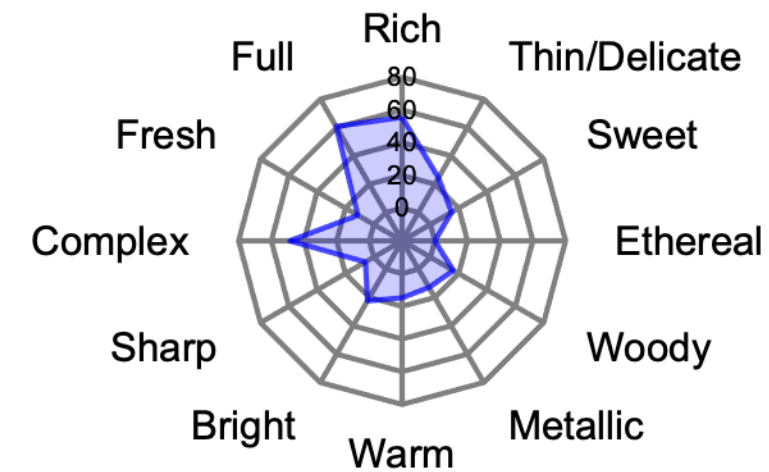

**Cherry**

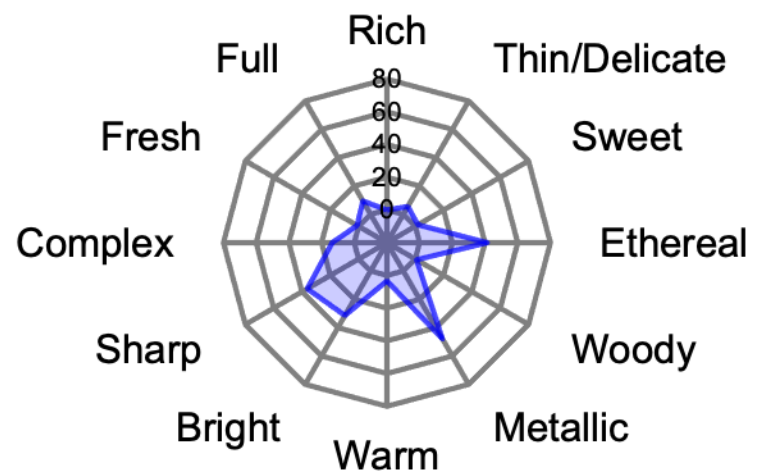

**Cinnamon**

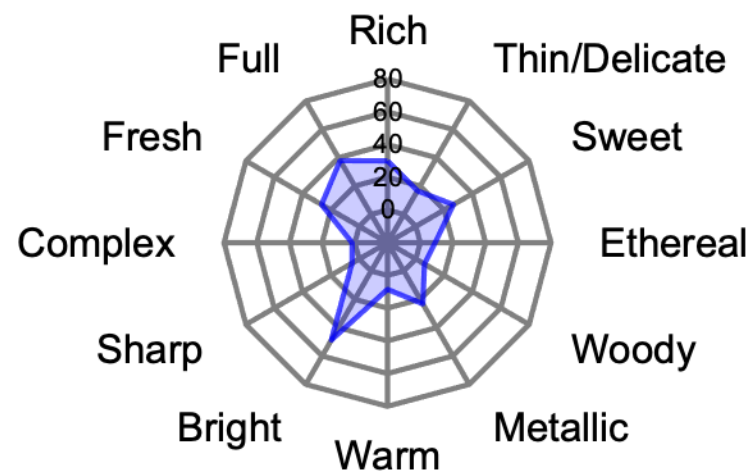

**Cocoa**

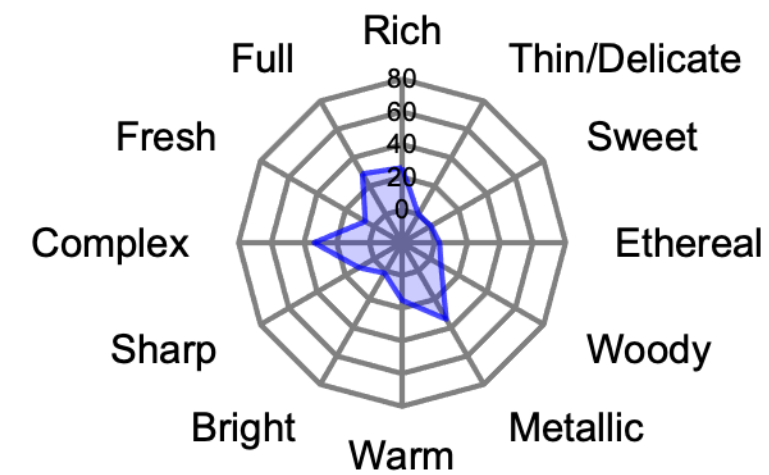

**Coffee**

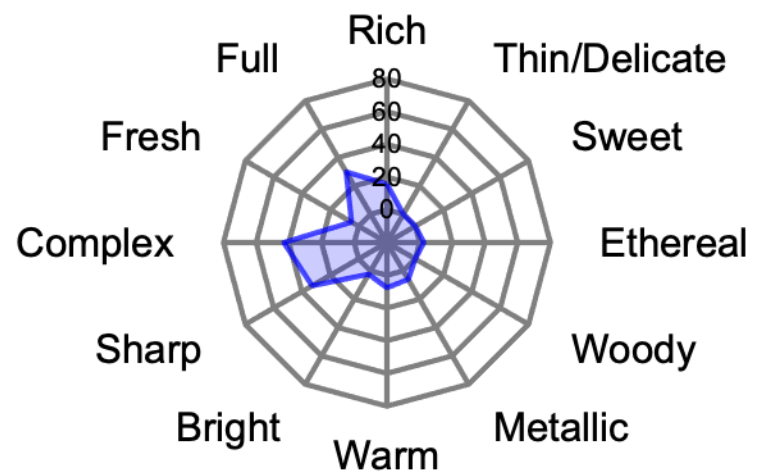

**Earthy**

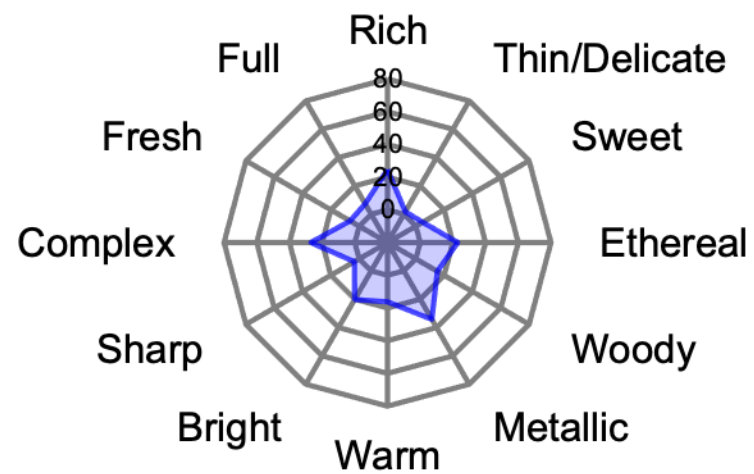

**Fruity**

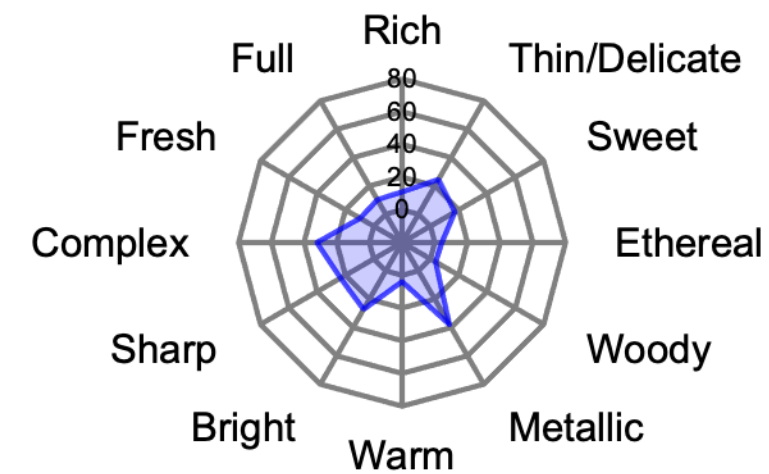

Supplement: Supplementary file 1 [file Data_Sheet_1.zip › Supplementary material_poetry_of_senses_2025/Figures/FigS1a.pdf]
